# Supplementary material for: Testing the advantages and disadvantages of short- and long- read eukaryotic metagenomics using simulated reads
Source: BMC Bioinformatics. 2020 May 29;21:220. doi: 10.1186/s12859-020-3528-4 (PMC7257156; doi:10.1186/s12859-020-3528-4)
Supplement: Supplementary file 1 — Additional file 1: Figure S1. The number of species present in the NCBI RefSeq database has grown roughly exponentially over time. Figure S2. Recall at the family level. Figure S3. Classification success at the family level. Table S1. List of species include in the in silico mock community, with associated Kingdom and NCBI. [file 12859_2020_3528_MOESM1_ESM.pdf]

Supplementary Material

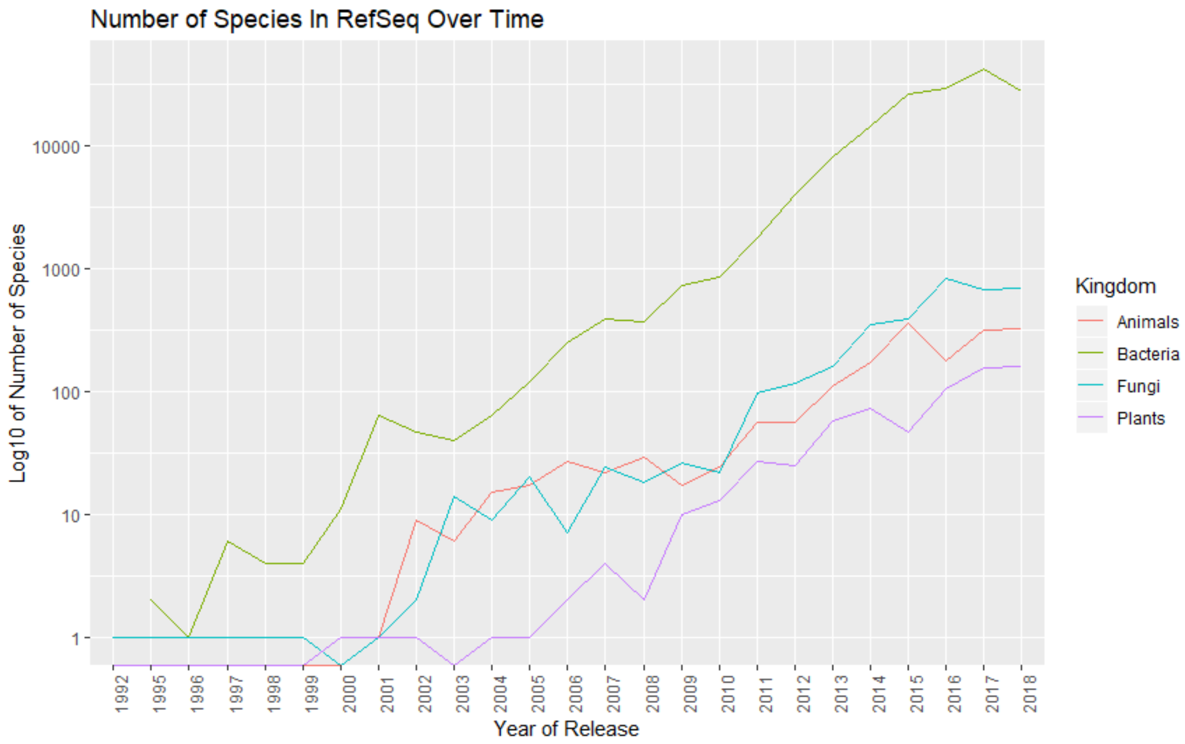

Supplementary Figure 1. The number of species present in the NCBI RefSeq database has grown roughly exponentially over time. Note that the y-axis is plotted on a log scale. Data were retrieved from the RefSeq database (47): https://ftp.ncbi.nlm.nih.gov/genomes/GENOME_REPORTS/eukaryotes.txt and https://ftp.ncbi.nlm.nih.gov/genomes/GENOME_REPORTS/prokaryotes.txt

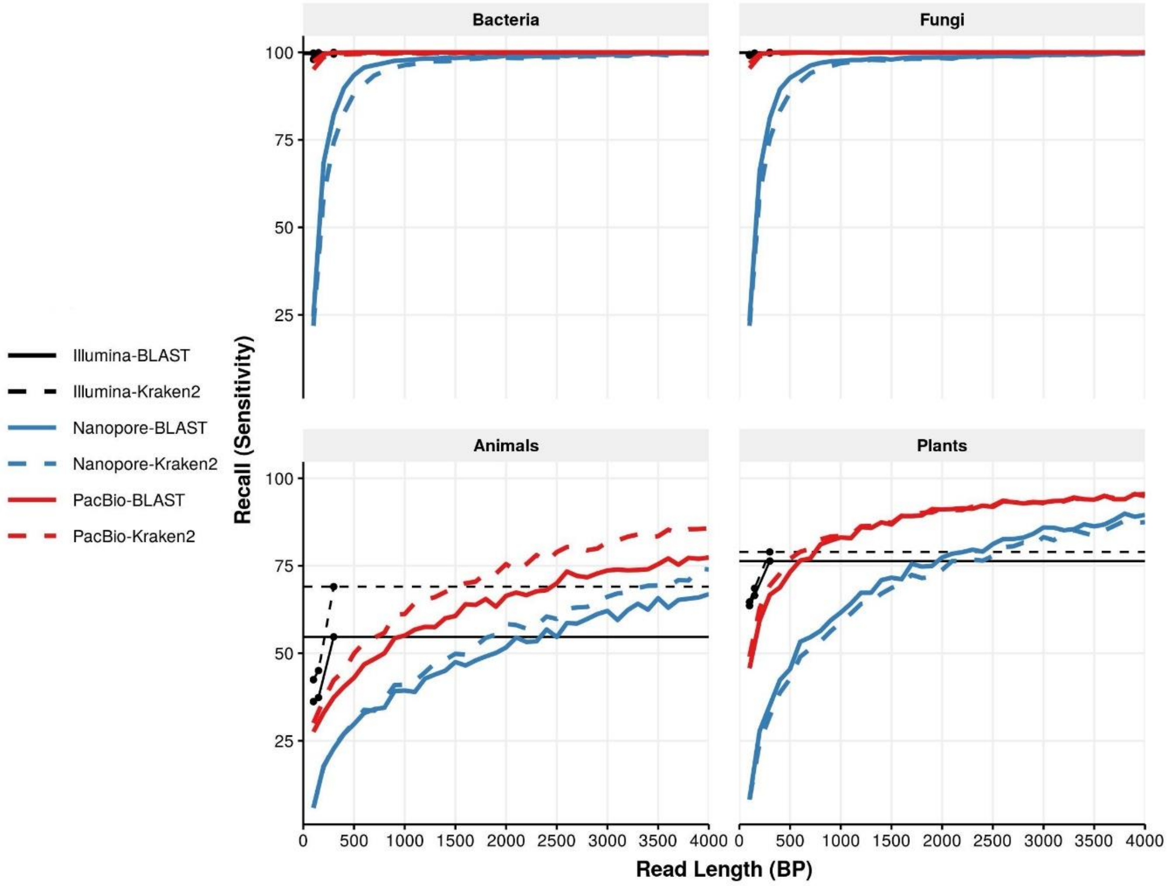

Supplementary Figure 2 Recall at the family level. Each panel shows recall for the different kingdoms. Median recall at the family level is indicated by blue lines for Nanopore or red lines for PacBio, either dashed (Kraken2) or solid (BLAST). The recall rates for 300 bp Illumina reads are shown as thin solid black lines, again either dashed (Kraken2) or solid (BLAST). Black points show the recall for all Illumina reads of all lengths (100 bp, 150 bp, and 300 bp).

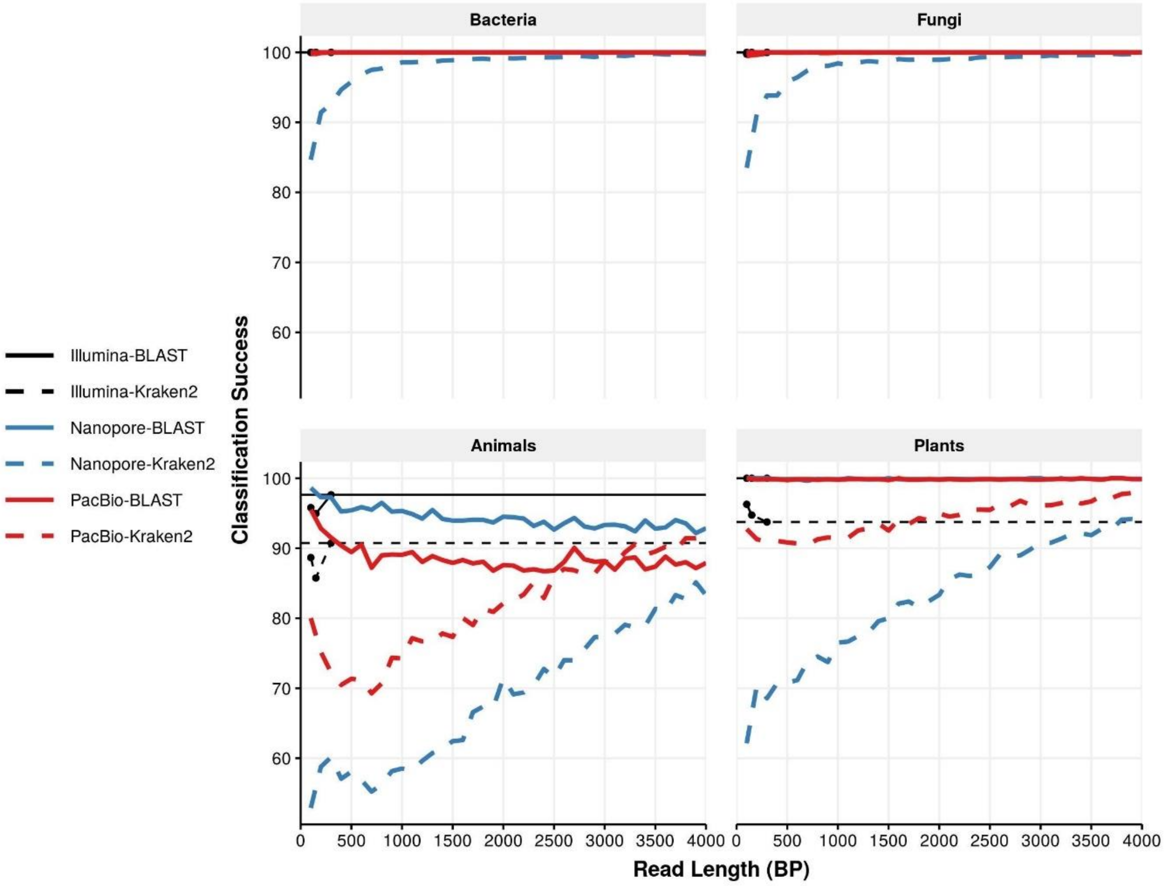

Supplementary Figure 3 Classification success at the family level. Each panel shows classification success for the different kingdoms. Median classification success at the family level is indicated by blue lines for Nanopore or red lines for PacBio, either dashed (Kraken2) or solid (BLAST). The recall rates for 300 bp Illumina reads are shown as thin solid black lines, again either dashed (Kraken2) or solid (BLAST). Black points show the classification success for all Illumina reads of all lengths (100 bp, 150 bp, and 300 bp). For animals and plants, the classification success of Kraken2 depends strongly on read length, and only surpasses BLAST in PacBio at approximately 3600bp.

Supplementary Table 1. List of species include in the *in silico* mock community, with associated Kingdom and NCBI

Species	Kingdom	NCBI Accession
Actinidia chinensis	Plantae	CM009654.1
Ananas comosus	Plantae	CM003813.1

Arabidopsis thaliana	Plantae	CP002684.1
Brassica nigra	Plantae	CM004491.1
Camelina sativa	Plantae	CM002729.1
Citrus sinensis	Plantae	CM001701.1
Dioscorea rotundata	Plantae	BDMI01000001.1
Eutrema salsugineum	Plantae	CM001778.1
Gossypioides kirkii	Plantae	CM008980.1
Leersia perrieri	Plantae	CM002476.1
Malus domestica	Plantae	CM007867.1
Micromonas sp.	Plantae	CP001574.1
Panicum hallii	Plantae	CM008046.2
Raphanus sativus	Plantae	CM007999.1
Rosa chinensis	Plantae	CM009582.1
Setaria italica	Plantae	CM004364.1
Solanum lycopersicum	Plantae	CM001064.3
Sorghum bicolor	Plantae	CM000760.3

Theobroma cacao	Plantae	LT594788.1
Trifolium pratense	Plantae	LT555306.1
Amphiprion percula	Animalia	CM009708.1
Bos indicus	Animalia	CM003021.1
Capra hircus	Animalia	CM001710.2
Chrysemys picta	Animalia	CM002655.1
Columba livia	Animalia	CM007525.1
Cyprinus carpio	Animalia	LN590701.1
Drosophila busckii	Animalia	CP012523.1
Equus caballus	Animalia	CM000377.2
Falco peregrinus	Animalia	CM007505.1
Homo sapiens	Animalia	CM004593.1
Lycaon pictus	Animalia	CM007565.1
Macaca mulatta	Animalia	CM000308.1
Microcebus murinus	Animalia	CM007661.1
Mus musculus	Animalia	CM004154.1

Oncorhynchus tshawytscha	Animalia	CM009202.1
Oryctolagus cuniculus	Animalia	CM000790.1
Ovis aries	Animalia	CM008472.1
Takifugu rubripes	Animalia	HE602535.1
Timema cristinae	Animalia	CM007794.2
Xiphophorus maculatus	Animalia	CM008938.1
Agaricus bisporus	Fungi	CP015470.1
Alternaria solani	Fungi	CP022024.1
Colletotrichum higginsianum	Fungi	CM004455.1
Cryptococcus gattii	Fungi	CP025759.1
Debaryomyces hansenii	Fungi	CR382133.2
Eremothecium sinecaudum	Fungi	CP014242.1
Flammulina velutipes	Fungi	CM002695.1
Fusarium verticillioides	Fungi	CM000578.1
Kluyveromyces lactis	Fungi	CR382121.1
Komagataella phaffii	Fungi	LT962476.1

Lachancea nothofagi	Fungi	LT598449.1
Malassezia sympodialis	Fungi	LT671813.1
Millerozyma farinosa	Fungi	FO082059.1
Ogataea parapolymorpha	Fungi	CM002300.1
Saccharomyces cerevisiae	Fungi	BK006935.2
Sporisorium scitamineum	Fungi	CP010913.1
Trichoderma reesei	Fungi	CP016232.1
Valsa mali	Fungi	CM003098.1
Yarrowia lipolytica	Fungi	HG934059.1
Zygosaccharomyces rouxii	Fungi	CU928173.1
Acidithiobacillus ferrivorans	Bacteria	LT841305.1
Bacillus thuringiensis	Bacteria	CP015250.1
Bacillus velezensis	Bacteria	CP025939.1
Bifidobacterium longum	Bacteria	CP013673.1
Bordetella bronchiseptica	Bacteria	CM002881.1
Brucella melitensis	Bacteria	CP018494.1

Campylobacter jejuni	Bacteria	CP012689.1
Caulobacter crescentus	Bacteria	AE005673.1
Cellvibrio japonicus	Bacteria	CP000934.1
Escherichia albertii	Bacteria	AP014855.1
Gordonibacter sp.	Bacteria	LT827128.1
Klebsiella pneumoniae	Bacteria	CP025088.1
Mycobacterium tuberculosis	Bacteria	CP023640.1
Ornithobacterium rhinotracheale	Bacteria	CP006828.1
Pseudomonas arsenicoxydans	Bacteria	LT629705.1
Salmonella enterica	Bacteria	CP007400.2
Serratia symbiotica	Bacteria	LN890288.1
Staphylococcus aureus	Bacteria	CP012974.1
Treponema pallidum	Bacteria	CP020366.1
Vibrio cholerae	Bacteria	LT907989.1
